# The Role of Perceived Speed in Vection: Does Perceived Speed Modulate the Jitter and Oscillation Advantages?

**DOI:** 10.1371/journal.pone.0092260

**Published:** 2014-03-20

**Authors:** Deborah Apthorp, Stephen Palmisano

**Affiliations:** 1 Research School of Psychology, Australian National University, Canberra, Australian Capital Territory, Australia; 2 School of Psychology, University of Wollongong, Wollongong, New South Wales, Australia; University of Sussex, United Kingdom

## Abstract

Illusory self-motion (‘vection’) in depth is strongly enhanced when horizontal/vertical simulated viewpoint oscillation is added to optic flow inducing displays; a similar effect is found for simulated viewpoint jitter. The underlying cause of these oscillation and jitter advantages for vection is still unknown. Here we investigate the possibility that perceived speed of motion in depth (MID) plays a role. First, in a 2AFC procedure, we obtained MID speed PSEs for briefly presented (vertically oscillating and smooth) radial flow displays. Then we examined the strength, duration and onset latency of vection induced by oscillating and smooth radial flow displays matched either for simulated or perceived MID speed. The oscillation advantage was eliminated when displays were matched for perceived MID speed. However, when we tested the jitter advantage in the same manner, jittering displays were found to produce greater vection in depth than speed-matched controls. In summary, jitter and oscillation advantages were the same across experiments, but slower MID speed was required to match jittering than oscillating stimuli. Thus, to the extent that vection is driven by perceived speed of MID, this effect is greater for oscillating than for jittering stimuli, which suggests that the two effects may arise from separate mechanisms.

## Introduction

Optic flow across the retina has been shown to be an important cue for humans and animals to navigate their way through the environment [1]–[4]. Vection is the experience in which a purely visual optic flow stimulus can induce a strong illusion of self-motion in a stationary observer [5]–[7]. An often-cited example is the train illusion, where observers seated on a stationary train view a train on the adjacent track beginning to move, and experience an illusion of self-motion in the opposite direction [8]. It has been shown that adding horizontal/vertical simulated viewpoint oscillation to radial optic flow displays increases vection in depth strength ratings, decreases vection onset latency, and increases vection durations [9]–[11]. Similar vection enhancements are found for simulated viewpoint jitter, which also strengthens the vection aftereffect (VAE) [12]–[16]. These types of global alterations to optic flow are similar to ecological situations which occur in everyday life - global oscillation of expanding optic flow occurs while walking at a regular pace, and jitter occurs while, for instance, riding a bicycle over rough terrain.

A complete explanation for jitter and oscillation advantages in vection is still elusive, and it is likely that there may be several factors involved. One possibility is that jittering/oscillating optic flow displays are more ‘ecological’ than smooth displays, as they are similar to the movement profile of real optic flow across the retina while walking [10], [17], [18]. Despite the intuitive appeal of this idea, the multiple elements involved in rendering displays more ‘ecological’, while holding low-level visual factors constant, make it a challenging hypothesis to test. Some researchers report that making a display more similar to the optic flow generated by real/natural self-motions improves vection. Bubka and Bonato [19] found that adding colour to self-motion displays (compared to black-and-white) and showing movies filmed while walking with a handheld camera (compared to those shot from a rolling cart) both improved vection ratings.

Another possible factor is reduced local motion adaptation [20]; in smooth radial flow displays, observers should experience adaptation to the optic flow stimuli, which should reduce the vection experienced over time [21]–[23]. However, adding both jitter and oscillation to these smoothly-moving displays would be expected to reduce the degree of adaptation. In support of this account, Seno et al. [9] found both reduced motion aftereffects (MAE) and increased vection for jittering and oscillating displays compared to smooth radial-flow displays. This account would also predict that vection from smooth radial flow should decline over time in comparison to jittering and oscillating radial flow, which is not generally found to be the case (at least with typical optic flow exposures of 30–60s). It should be pointed out, however, that many vection studies rely on discrete rather than continuous measures of vection, such as vection strength ratings, latency and duration.

Increased global retinal motion has also been suggested as another possible mechanism [12], [24]–[26]. Adding horizontal/vertical simulated viewpoint jitter or oscillation to 3D radial flow should increase the observer's global retinal motion irrespective of whether he/she maintains a stable gaze or pursues moving objects in the self-motion display. When the observer maintains stable fixation, then all of the jitter or oscillation will be added to his/her retinal flow. During free viewing, though the eyes will track oscillating stimuli to increase retinal stability, this tracking is imperfect and adapts over time [24], leading to increases in total retinal motion for both jittering and oscillating stimuli. In keeping with this hypothesis, Palmisano et al. [27] found that both display oscillation and fixation point oscillation increased linear vection compared to smooth radial flow displays viewed centrally. In addition, Palmisano, Kim and Freeman [27] found that the ‘slalom illusion’, where observers tracked an oscillating fixation point while viewing smooth frontal-plane motion, produced equivalent increases in vection to conditions where the fixation point was stationary and the display oscillated. This was recently replicated for vertical vection [26]. It should be noted that increased global retinal motion may not always lead to increased vection: Palmisano et al. [28] found that adding simulated unidirectional (horizontal/vertical) *constant-velocity* linear self-motion to radial flow did not result in vection increases, even though it would have increased global retinal motion compared to purely radial flow. This suggests that the accelerating/decelerating profiles of the simulated viewpoint jitter and oscillation also play an important role in generating these vection advantages.

It is important to note here that there are several different aspects of this global retinal motion increase that could be important in self-motion perception. Firstly, the jitter and oscillation components of the motion could increase the amount of relative motion seen in the display - that is, the motion of each component relative to that of other components. Second, the additional motion components increase the absolute speed of the display (as opposed to the MID speed). Relative motion is well established as a more compelling cue to motion direction [29] and speed [30]–[32] than absolute motion. There are several types of relative motion that are relevant to direction perception: the motion of objects relative to one another, the motion of objects relative to a frame of reference, and the relative motion of objects from one moment to another, i.e. variations in speed over time. If the observer makes a judgment of MID speed that is to some extent dependent on the relative display speed, then this might increase the perceived speed of MID, which may in turn result in increased vection ratings.

In further evidence that speed and vection magnitude are related, it has been shown that increasing *stimulus* (or retinal) speed can increase vection [7], [33], [34]. In addition, other display manipulations which increase perceived speed can also result in increased vection. Examples of this include adding consistent stereoscopic cues to the optic flow [35], [36], increasing display size [37], and increasing stimulus spatial frequency in central vision while decreasing it in peripheral vision [38]. Wist et. al. [39] also found that the *speed* of circular vection was increased by increasing the perceived distance of the inducing drum using the Pulfrich effect, thus increasing perceived speed while leaving retinal (linear) speed constant. The authors did not record vection magnitude, but vection magnitude and vection speed have been argued to be closely related [13].

The jitter and oscillation advantages seem remarkably similar in many respects, even though the frequency profiles of the motion are quite different. Are the two effects underpinned by the same mechanisms? Previous research has shown that adding randomly-generated simulated viewpoint jitter produces an equivalent [28] or greater [9] increase in vection compared to simulated viewpoint oscillation; however, studies comparing the effects of oscillation and jitter on vection often suggest the two may be tapping at least partly separate mechanisms. For instance, Seno, Palmisano and Ito [9] found increases in vection aftereffects for jittering but not for oscillating stimuli (compared to smooth radial flow), although both resulted in reduced motion aftereffects. If jittering radial flow displays tap a separate (or at least partially separate) mechanism of self-motion perception, perhaps at a higher level of the visual system, then we may see diverging effects of perceived speed.

Here we aim to explore the role of perceived speed in both the jitter and oscillation advantages in vection. Do oscillation and/or jitter increase the perceived speed of MID (for example, by increasing the amount of relative motion in the display), and if so, can this perceived speed increase account for the advantages of these manipulations on the vection experience? If perceived (rather than simulated) speed of MID is important in determining vection, then a smoothly-moving display that is matched to the same perceived speed as an oscillating or jittering display should produce the same amount of vection. Alternatively, if the underlying mechanisms of jitter and oscillation advantages differ, we may find that perceived speed plays more of a role in one than the other. In addition, the role of adaptation can be addressed by the use of a continuous measure of vection strength; if reduced adaptation is a factor in the jitter and oscillation advantages, then jittering and oscillating displays should produce less decline in vection ratings over time compared to smooth radial flow. We will also examine the role of longer-term speed adaptation by analysing vection ratings across trials during the experiments.

## Results

### Statistical analyses

All statistical analyses reported below were carried out in SPSS Version 21 for Macintosh. Unless otherwise reported, all analyses are repeated-measures ANOVAs and paired t-tests, corrected where necessary for multiple comparisons. All assumptions for ANOVA were met, and, where appropriate, Greenhouse-Geisser corrections for departures from sphericity were used.

### Experiment 1.1: Speed comparison for oscillating stimuli

**Figure 1:**
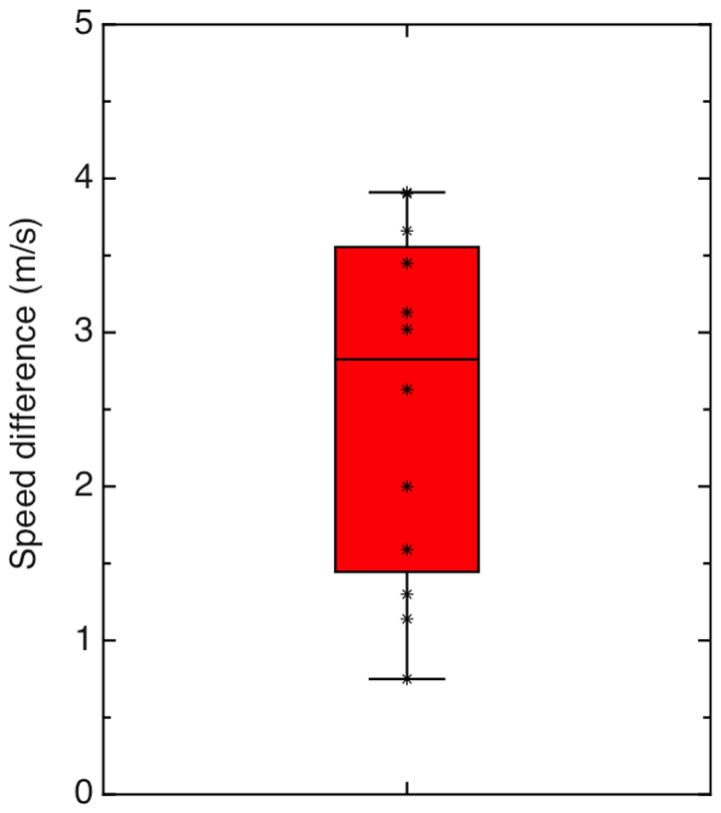
Results for Experiment 1.1. This box-and-whisker plot illustrates the increase in the simulated MID speed required to match the perceived MID speed of the oscillating optic flow stimuli. The coloured area shows the central two quartiles, bisected by the median, and asterisks show individual data points.

In a two-interval, forced-choice experiment, participants matched the perceived speed of smoothly-moving radial flow displays to that of vertically oscillating radial flow displays, with the speed of the comparison display adjusted in an adaptive staircase procedure to reach the point of subjective equality (PSE) - see Methods for details. Display durations (1 sec each) were deliberately chosen to be too brief to induce vection [7]. The MID speeds selected to match the smooth with the oscillating radial flow were higher than the simulated MID speeds of the oscillating displays for every participant; the mean increase in MID speed required to match the perceived speed of smooth with oscillating stimuli was 2.54 m/s, t(11)  = 7.72, p<0.0001. Since the baseline simulated speed was 4 m/s, this represents an increase in perceived speed of 63%, on average. Results are illustrated in Figure 1. These individually-matched speeds were then used in Experiment 1.2 for longer time periods to induce vection, along with oscillating and radial flow displays which had the same simulated MID speed (4 m/s).

### Experiment 1.2: Vection for smooth, oscillating and speed-matched stimuli

In an experiment comprising longer sessions of motion stimuli, participants viewed 30-second radial motion displays which simulated either smooth or vertically oscillating motion-in-depth (randomly interleaved); the smooth motion was either at the same simulated MID speed as the oscillating motion (4 m/s) or matched to the individually-obtained perceived speed of the oscillating display. Participants rated vection continuously with a throttle device (previously used in other studies to give a continuous measure of vection; see [10], [24]), and also gave verbal ratings after each vection display (see Methods for details).

**Figure 2:**
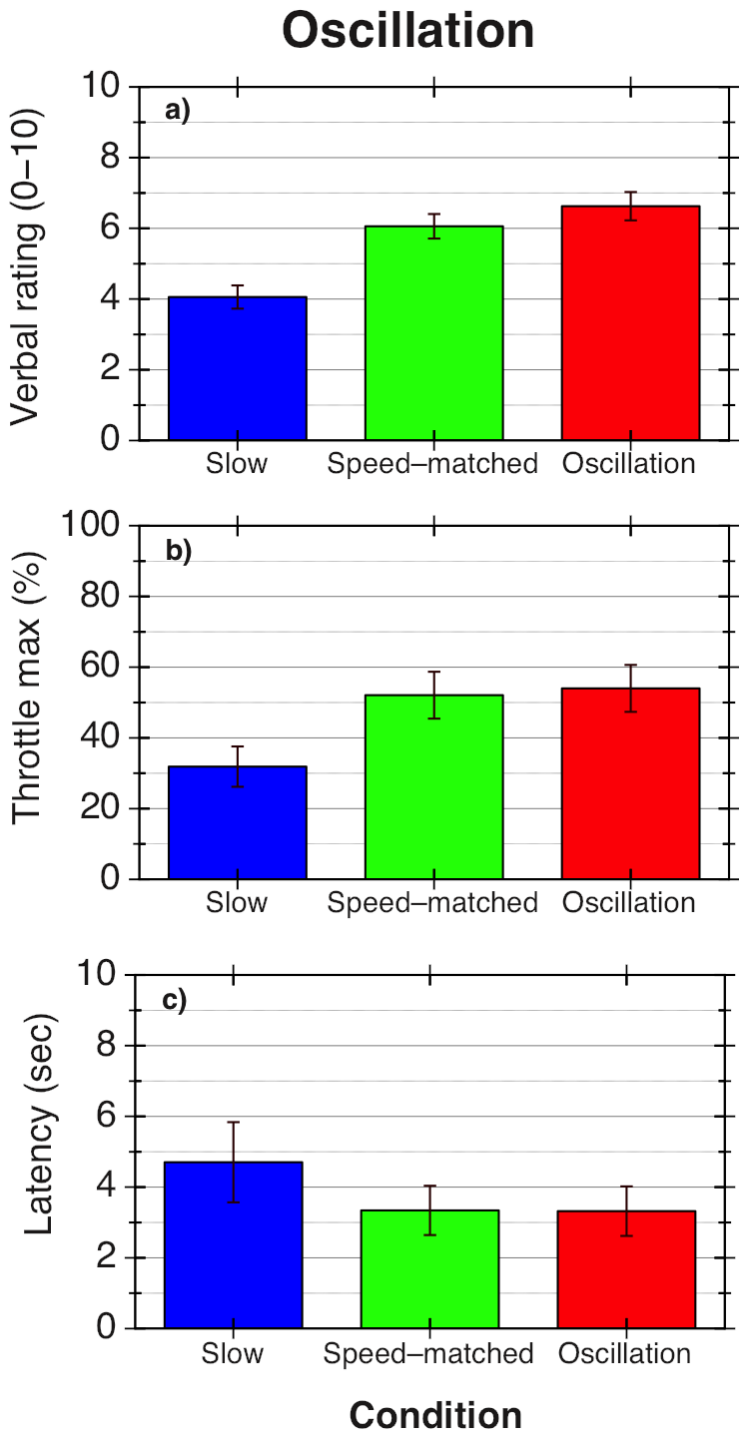
Results for Experiment 1.2. Results are averaged over 12 participants: a) Mean verbal vection magnitude ratings (0–10) for each stimulus type, averaged for each participant over 8 trials; b) mean values for the maximum throttle setting reached, as a percentage of the total possible range; c) mean vection onset latency, measured in seconds, as given by the time taken for the throttle to be moved to a cutoff value of 5% of the maximum value. Error bars show ±1 standard error in all graphs.

Overall, there were strong increases in both verbal and throttle-based (i.e. maximum throttle setting) vection magnitude ratings for the oscillating displays (relative to the ‘slow’ smooth radial flow displays). There was also a significant decrease in the vection latencies for these oscillating displays. However, there was no significant difference on any vection measure between the smooth speed-matched and oscillating displays. Results are presented in Figure 2. The main effect of condition (slow smooth, fast smooth and oscillating) was significant across all vection measures: for verbal magnitude ratings, F(2, 22)  = 16.77, p<0.001; for maximum throttle settings, *F*(2, 22) = 20.53, p<0.001; and for latencies, *F*(2, 22)  = 4.09. p = 0.031. The results of post hoc tests for each measure are shown in Table 1. In brief, there were no differences between oscillating and speed-matched conditions for any of the measures (p>0.5); both oscillating and speed-matched conditions produced significantly higher verbal magnitude ratings and maximum throttle values than the slow-speed smooth radial flow condition. None of the differences in latency were significant after correcting for multiple comparisons.

**Table 1:** Results of post-hoc tests between conditions for each vection measure.

	O vs. SM	O vs. S	SM vs. S
Verbal (1–10)	1.037 (>.5)	5.764 (<0.001)	5.130 (0.001)
Throttle max (%)	.493 (.5)	6.072 (0.001)	15.045 (0.001)
Latency (s)	.065 (.5)	2.045 (.195)	2.285 (.129)

p-values are in brackets, Bonferroni-corrected for multiple comparisons. O refers to oscillating conditions, S to slow, and SM to speed-matched.

**Figure 3:**
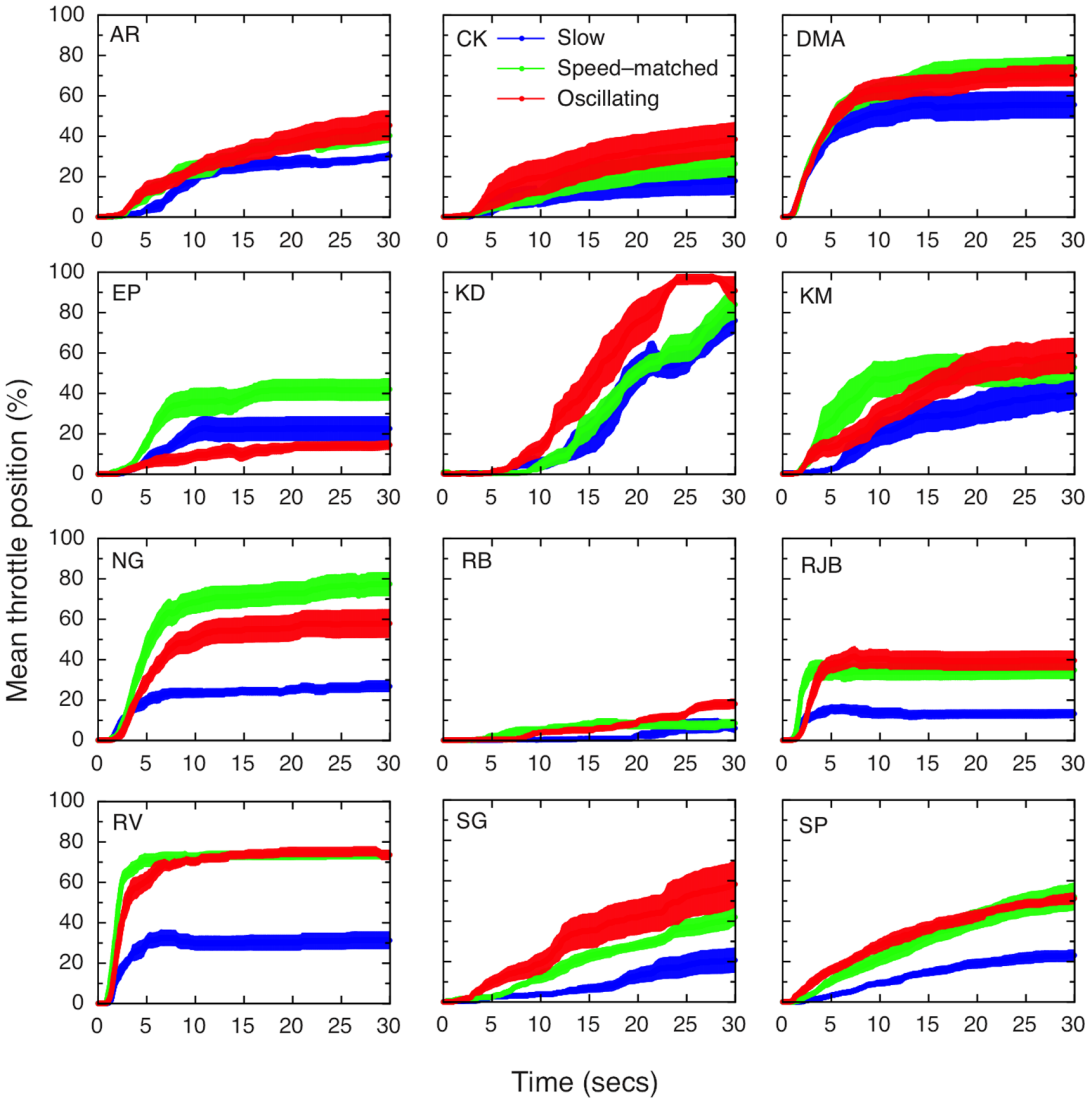
Individual throttle traces for each participant over the three conditions, averaged across 8 trials. It is clear that, for the majority of the participants, the speed-matched (green) and oscillating (red) stimuli produced very similar throttle ratings. The position of the throttle device was sampled at 100 Hz, the same as the frame rate, and participants reset the device to the zero position in between trials. Shaded areas show 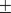
1 standard error of the mean throttle position at each sampled location over the 8 trials.

Figure 3 shows the throttle traces for each participant, averaged across the eight trials for each stimulus type. Although there is substantial variation in the levels of vection reported, participants' maximum throttle settings on a trial-by-trial basis (n = 96) were highly correlated with their verbal ratings (slow: r = .655, p<0.001; speed-matched: r = .693, p<0.001; oscillating, r = .681, p<0.001), and it can be seen that, for the majority of participants, the traces for the stimuli which were matched for perceived speed (green lines) very closely follow the traces for the oscillating stimuli (red lines). It is also worth noting that there were no drop-outs (which would have resulted in dips later in the trials), even though participants were instructed that they could move the throttle back if they stopped experiencing vection. From the traces, it is clear that there are some reductions in the throttle based vection settings, indicating participants did realise that the throttle could also be used to express reductions as well as increases in vection. Interestingly, verbal magnitude ratings were also significantly negatively correlated with latency (that is, higher verbal ratings were associated with shorter latencies on a trial-by-trial basis) for speed-matched (r = −.259, p = 0.011) and oscillating, (r = −.349, p<0.001) conditions, but not for the slow condition (r = −.174, p = 0.09). Rather unsurprisingly (since it was also determined by the throttle), latency was also correlated with throttle maximum values for all three conditions (slow: r = −.307, p = 0.002; speed-matched: r = −.314, p = 0.002; oscillating: r = −.344, p = 0.001).

**Figure 4:**
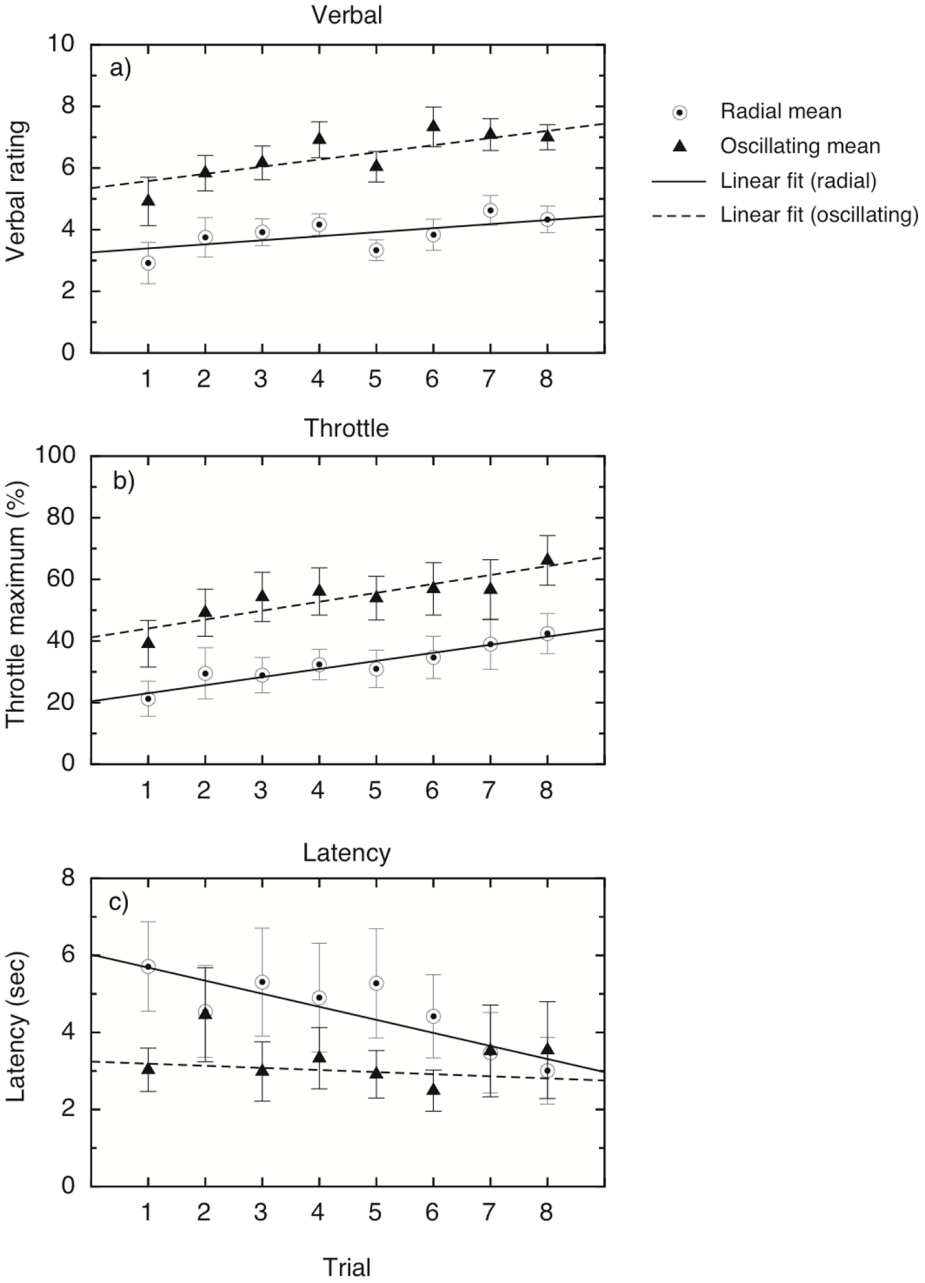
Trial-by-trial analysis of the ratings from Experiment 1.2. Results are averaged over 12 participants: a) Verbal vection ratings (0–10) for smooth (4 m/s) and oscillating (4 m/s) flow, shown over 8 trials; b) maximum throttle setting reached, as a percentage of the total possible range; c) mean vection onset latency, measured in seconds, as given by the time taken for the throttle to be moved to a cutoff value of 5% of the maximum value. Error bars show 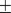
1 standard error in all graphs.

It is also interesting to note that none of the participants showed a decline in vection over the 30 s in any of the conditions, as evidenced by the absence of a decline in throttle values in any of the conditions. This seems to indicate that short-term motion adaptation was not a factor in any of the conditions. To examine whether there was any longer-term effect of adaptation on vection, we looked at the trial-to-trial variations in the three measures for each participant. In order to do this, we performed a two-way repeated-measures analysis on each of the vection measures for oscillating and smooth radial motion across trials, with trial and condition as factors (see Figure 4). Thus we were able to examine the effect of trial number (which would reveal a build-up or decline of vection across trials) and the effect of condition (smooth vs. oscillating motion) separately. If the two conditions differed in the amount of long-term adaptation, we would expect to see an interaction between condition and trial. For verbal ratings, there were significant main effects of condition, but no interaction between the effects (see Table 2 for the statistical figures). There was a significant linear trend for trial, showing an overall increase for vection ratings across trials (see Table 2). Similar results were found for the throttle maximum values; there were main effects of condition and trial, but no significant interaction, and again a significant linear trend for trial (see Table 2). None of the higher-order trends were significant for either measure. For the latency measure, neither main effect was significant, nor was the interaction or the linear trend. Overall, the results suggest a linear increase in vection as the session progressed, but this did not differ between smooth radial motion and oscillating motion. Thus, there is little support for the notion that reduced adaptation underlies the oscillation advantage in vection.

**Table 2:** F-statistics and p-values for the trial-by-trial analysis of Experiment 1.2.

Measure	Condition	Trial	Interaction	Trend
Verbal (1-10)	28.89 (.001)	4.68 (0.008)	1.27 (.3)	10.16 (.009)
Throttle max (%)	32.13 (.001)	3.97 (0.012)	0.26 (.5)	10.52 (.008)
Latency (s)	4.45 (.059)	1.1 (.361)	2.44 (.055)	2.66 (.131)

Degrees of freedom for the main effect of condition and for the linear trend are (1,11), and for the main effect of trial and the interaction between trial and condition are (7,77). All p-values are corrected via the Greenhouse-Geisser method to adjust for departures from sphericity where necessary. None of the higher-order trends were significant.

### Experiment 2: Jitter

The previous experiment examined the effect of perceived speed on vection by matching the simulated speed of a smoothly-moving radial flow stimulus to the perceived speed of a vertically oscillating stimulus, and found that the vection increases caused by oscillating stimuli could be completely matched by equating the perceived speeds of the two stimuli. Here we repeated Experiment 1 with vertically jittering, instead of oscillating, radial flow displays. Jittering stimuli moved at the same simulated MID speed as the oscillating stimuli described above, but instead of smooth sine-wave motion, the stimuli were shifted randomly in the vertical direction every 3 frames, over a range of approximately 0–10 Hz - see Methods for details.

### Experiment 2.1: Speed comparison for jittering stimuli

Experiment 2.1, as described above, asked subjects to compare the MID speeds of vertically jittering and smooth radial motion displays with brief durations (1 sec each, too brief to produce vection). PSEs (points of subjective equality) were determined as above. The MID speeds selected to match those for the jittering stimuli were higher for all but two participants; the mean increase in simulated MID speed required to match the perceived MID speed of the jittering stimuli was 1.15 m/s, t(9)  = 2.9, p = 0.017. This represents a perceived speed increase of 29% (compared to the baseline speed of 4 m/s). Interestingly, comparing this to the results of Experiment 1.1 in an independent two-sample t-test showed that the simulated MID speed increase required to match jittering stimuli was significantly lower, t(20)  = 2.735, p = 0.013.

### Experiment 2.2: Vection for smooth, jittering and speed-matched stimuli

**Figure 5:**
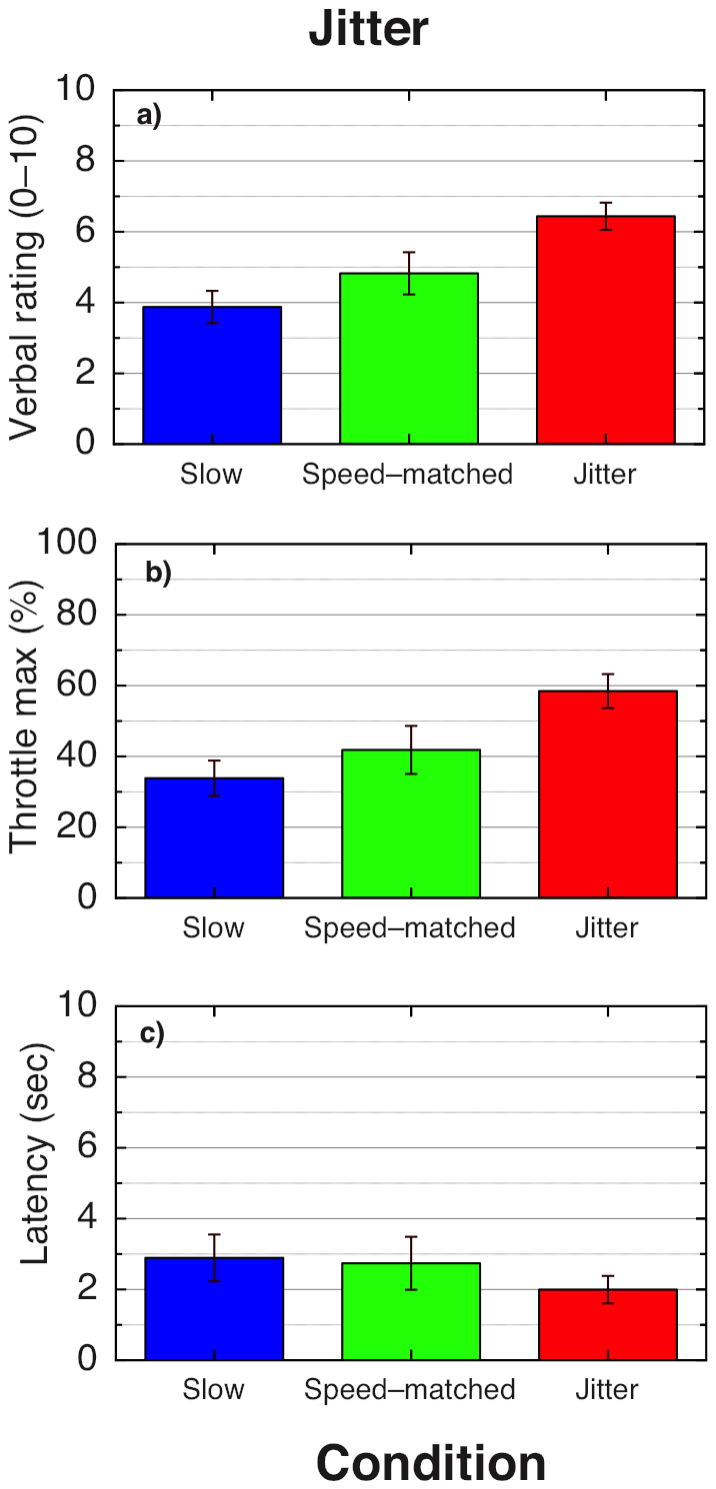
Results for Experiment 2.2. Results are averaged over 10 participants: a) Mean verbal ratings (0–10) for each stimulus type, averaged for each participant over 8 trials; b) mean values for the maximum throttle value reached, as a percentage of the total possible range; c) mean latency, measured in seconds, as given by the time taken for the throttle to be moved to a cutoff value of 5% of the maximum value. Error bars show 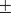 1 standard error in all graphs.

**Figure 6:**
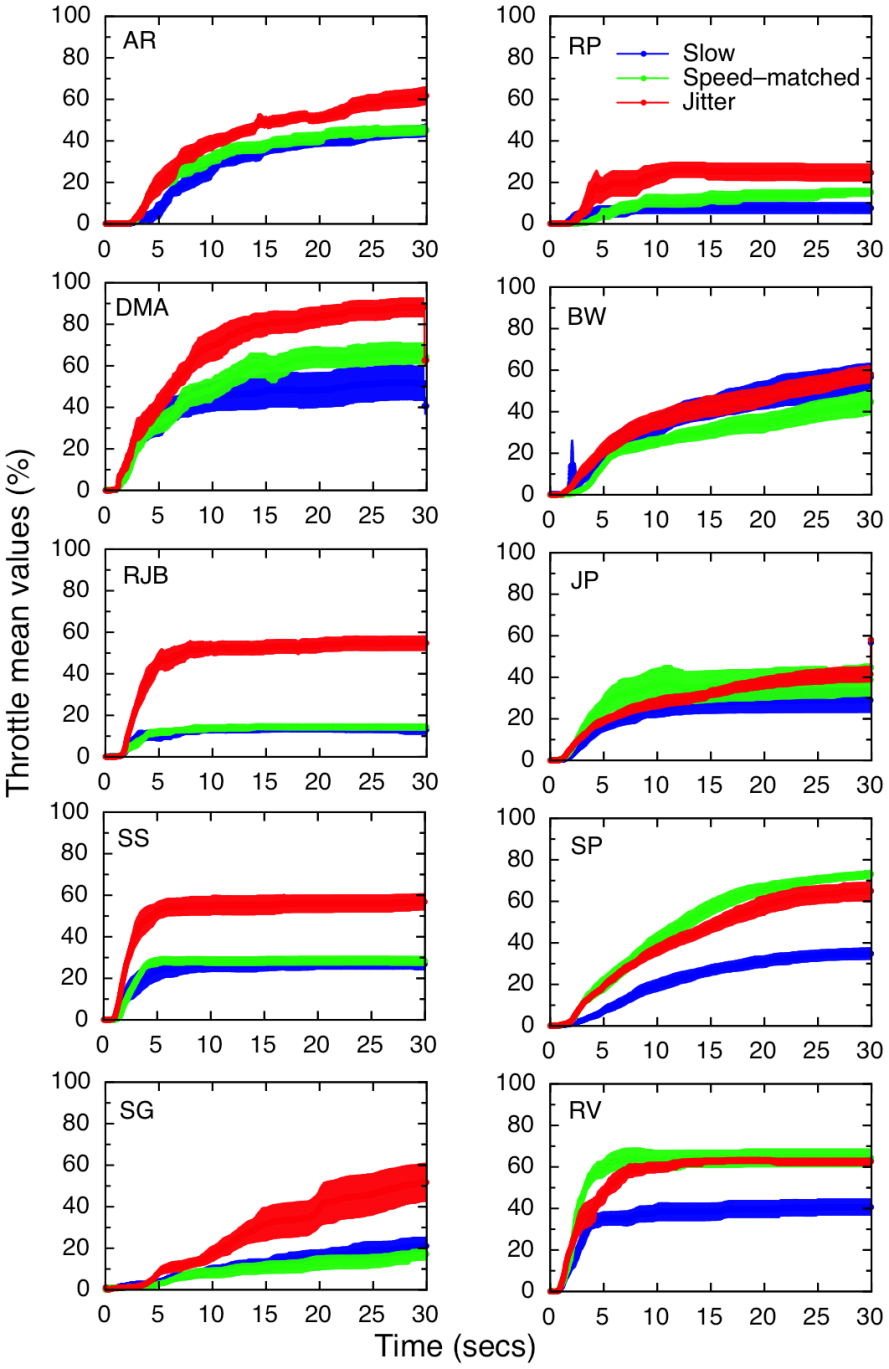
Throttle traces for individual participants. Mean throttle trace across 8 trials for each stimulus type for each of the 10 participants in Experiment 2.2. Shaded areas show 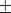
1 standard error of the mean throttle position at each sampled location over the 8 trials.

Experiment 2.2 was run exactly as described in Experiment 2.1, with the single exception that vertically jittering displays (as described in detail in the Methods section) were used in place of vertically oscillating displays. Again, there was a strong increase in verbal and throttle-based vection magnitude measures for the jittering stimuli, and a decrease in vection latency times; however, now there was no significant difference for any vection measure between the speed-matched and slow smooth stimuli, although there was a trend (p = .06) for stronger verbal vection magnitude ratings when comparing speed-matched and slow smooth stimuli. Mean results are presented in Figure 5, with individual throttle traces shown in Figure 6. The main effect of condition (slow, fast and oscillating) was significant across all measures: for verbal vection magnitude ratings, *F*(2, 18)  = 20.67, p<0.001; for maximum throttle ratings, *F*(2, 18)  = 15.01, p<0.001; and for vection latencies, *F*(2, 18)  = 3.62. p = 0.048. The results of post hoc tests for each vection measure are shown in Table 3. In summary, jittering displays produced significantly greater verbal vection ratings and maximum throttle settings than both the slow and speed-matched displays; for the latency measures, although there was a trend for shorter vection latencies when comparing jittering to slow displays (p = 0.068), none of the comparisons were significant after correcting for multiple comparisons.

**Table 3:** Results of post-hoc tests between conditions for each vection measure in Experiment 2.2.

	J vs. SM	J vs. S	SM vs. S
Verbal 1–10)	3.5 (.021)	6.43 (<.001)	2.82 (.06)
Throttle max (%)	3.2 (.033)	6.45 (.001)	1.72 (.357)
Latency (s)	2.31 (.141)	2.53 (.068)	.46 (.657)

p-values are in brackets, Bonferroni-corrected for multiple comparisons. J refers to jittering conditions, S to slow, and SM to speed-matched.

**Figure 7:**
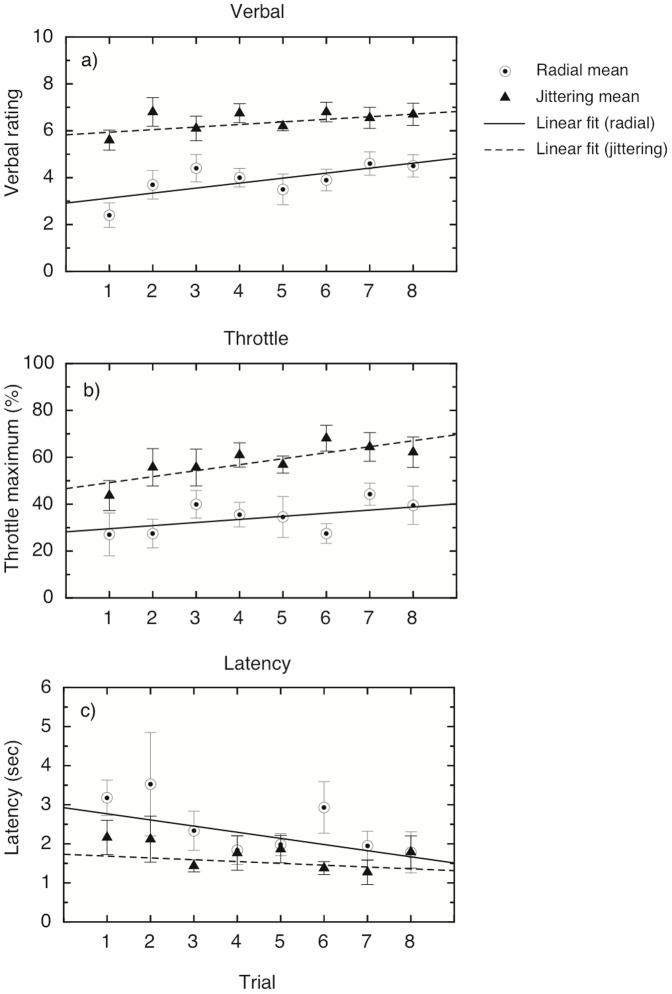
Trial-by-trial analysis of the ratings from Experiment 2.2. Results are averaged over 10 participants, and show the averages for each trial separately; linear regression fits are for illustrative purposes only. a) Verbal vection ratings (0–10) for smooth (4 m/s) and oscillating (4 m/s) flow, shown over 8 trials; b) maximum throttle setting reached, as a percentage of the total possible range; c) mean vection onset latency, measured in seconds, as given by the time taken for the throttle to be moved to a cutoff value of 5% of the maximum value. Error bars show 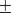
1 standard error in all graphs.

Again, looking at the throttle traces, it is clear that there were few or no drop-outs or reductions in vection during individual trials (see Figure 6). It is also clear that, for most of the participants, the trace for the speed-matched stimuli follows very closely the trace for the slow rather than the jittering stimuli. To investigate whether long-term adaptation played a role, as outlined above, we examined the three measures on a trial-by-trial basis, to look for any increase or decrease in vection across trials (see Figure 7). Two-way repeated-measures ANOVAs (with condition and trial as factors) showed that, for the verbal measures, there were again significant main effects of condition and trial, but no interaction; the main effect of trial showed a significant positive linear trend (see Table 4 for the statistical figures). The results were similar for the throttle measure, although the main effect for trial and the linear trend did not reach significance for this measure. For latency, the main effect for condition was significant, but not the effect for trial or the interaction; interestingly, the linear trend for trial was significant here, but this is difficult to interpret in the absence of a main effect.

**Table 4:** F-statistics and p-values for the trial-by-trial analysis of Experiment 2.2.

Measure	Condition	Trial	Interaction	Trend
Verbal (1–10)	41.36 (.001)	5.93 (.002)	2.58 (.071)	18.1 (.002)
Throttle max (%)	31.6 (.001)	2.78 (.069)	2.11 (.110)	5.01 (.052)
Latency (s)	6.39 (.032)	2.15 (.119)	0.875 (.428)	5.4 (.045)

Degrees of freedom for the main effect of condition and for the linear trend are (1,9), and for the main effect of trial and the interaction between trial and condition are (7,63). All p-values are corrected via the Greenhouse-Geisser method to adjust for departures from sphericity where necessary. Again, none of the higher-order trends were significant.

**Figure 8:**
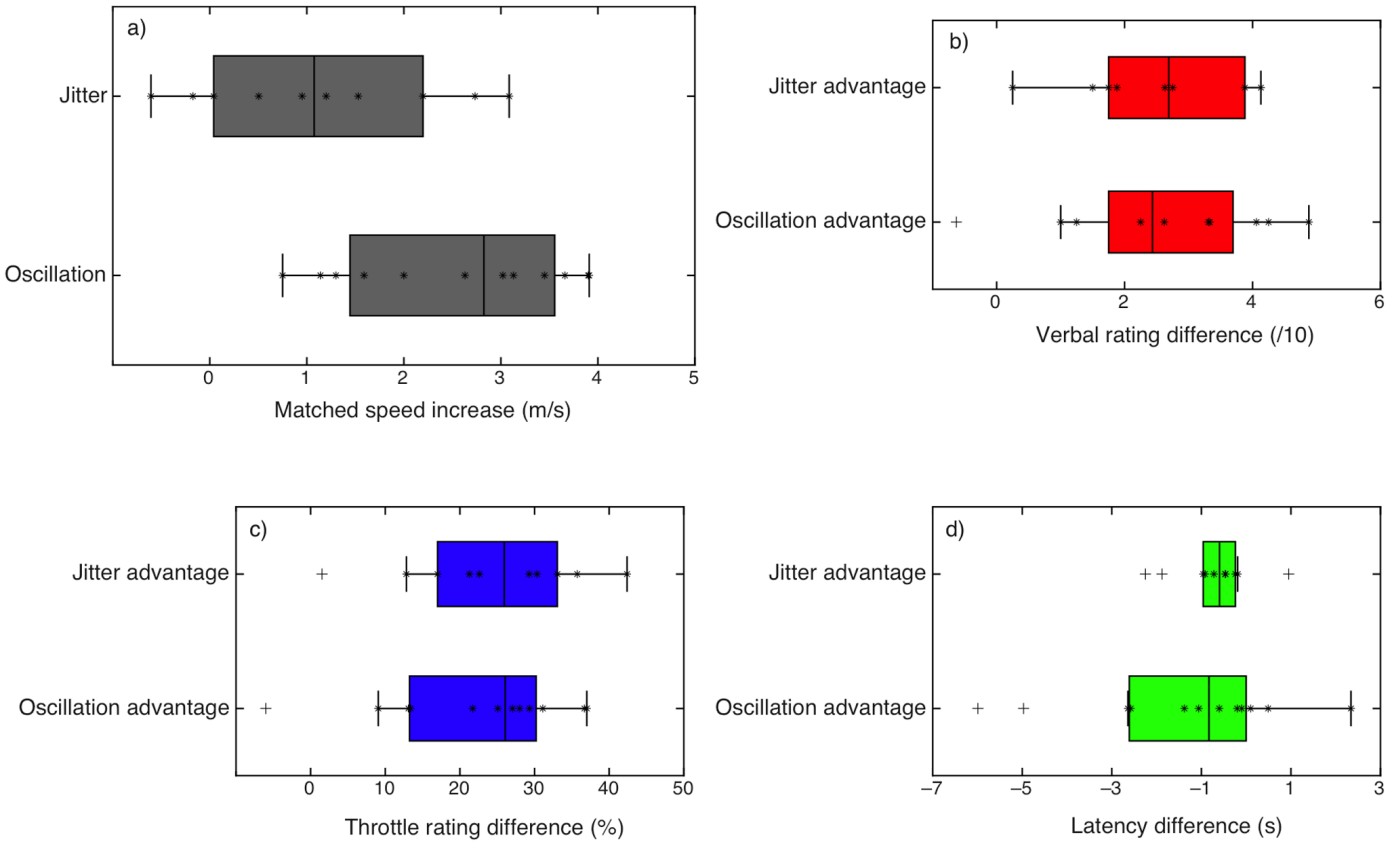
Comparison of perceived speed differences and vection advantages for oscillating and jittering stimuli. a) Speed increases required to match MID speed for jittering stimuli compared to smooth (top) were significantly less than those for oscillating stimuli. b) – d): Jitter and oscillation advantages for verbal (b), throttle maximum (c) and latency (d) did not differ significantly.

It is worth investigating whether the two display manipulations - oscillation and jitter - had different effects on vection in terms of the increase in vection ratings (see Figure 8). A set of independent two-sample t-tests between the *increase* in vection for oscillation and jitter (Experiments 1.2 and 2.2) showed no significant difference between the two display manipulations for any of the vection measures, in spite of the significant difference in perceived speed increase reported above (see Table 5), which suggests there was enough power to detect a difference in vection strength if it had been there. Previous reports show that jitter sometimes (but not always) produces greater increases in vection than oscillation [9], [12], [28]; these differences may depend somewhat on the stimulus parameters. While it could be argued that our sample sizes did not provide enough power to detect a difference, there was enough power to detect the difference in perceived speed (see Experiment 2.1 and Figure 8); power to detect a difference of 1.5 units in any of the vection measures (based on previous studies) was calculated at .81 for this sample size.

**Table 5:** Results of independent-samples t-tests between Experiments 1 and 2 for the jitter/oscillation advantage, for each of the vection measures.

	Difference (exp 1–2)	t (20)	p
Verbal (1–10)	0.007	0.011	0.991
Throttle max (%)	−2.474	0.467	0.646
Latency (s)	−1.115	0.849	0.406

### Experiment 3: Precision of speed discrimination for motion-in-depth

Experiment 3 was run to examine the possibility that the differing effects of perceived speed were due to the fact that participants had more difficulty judging the speed of MID for some conditions than for others - for instance, erroneously misattributing some of the vertical motion of oscillation to MID. This difficulty would result in a loss of precision for discriminations between MID speeds for those types of stimuli. Thus we asked participants, as in Experiments 1.1 and 2.1, to discriminate MID stimuli, but this time for the same stimuli (smooth MID, oscillating MID, and jittering MID at the simulated speed of 4 m/s, and also smooth MID at the simulated speed of 6 m/s, the average of the faster speeds used). As in the earlier experiments, we used QUEST adaptive staircases to estimate the thresholds for discriminating these stimuli, but this time, rather than the PSE (point of subjective equality), we were interested in the slope (beta), which represents the standard deviation or precision for discriminating the stimuli.

**Figure 9:**
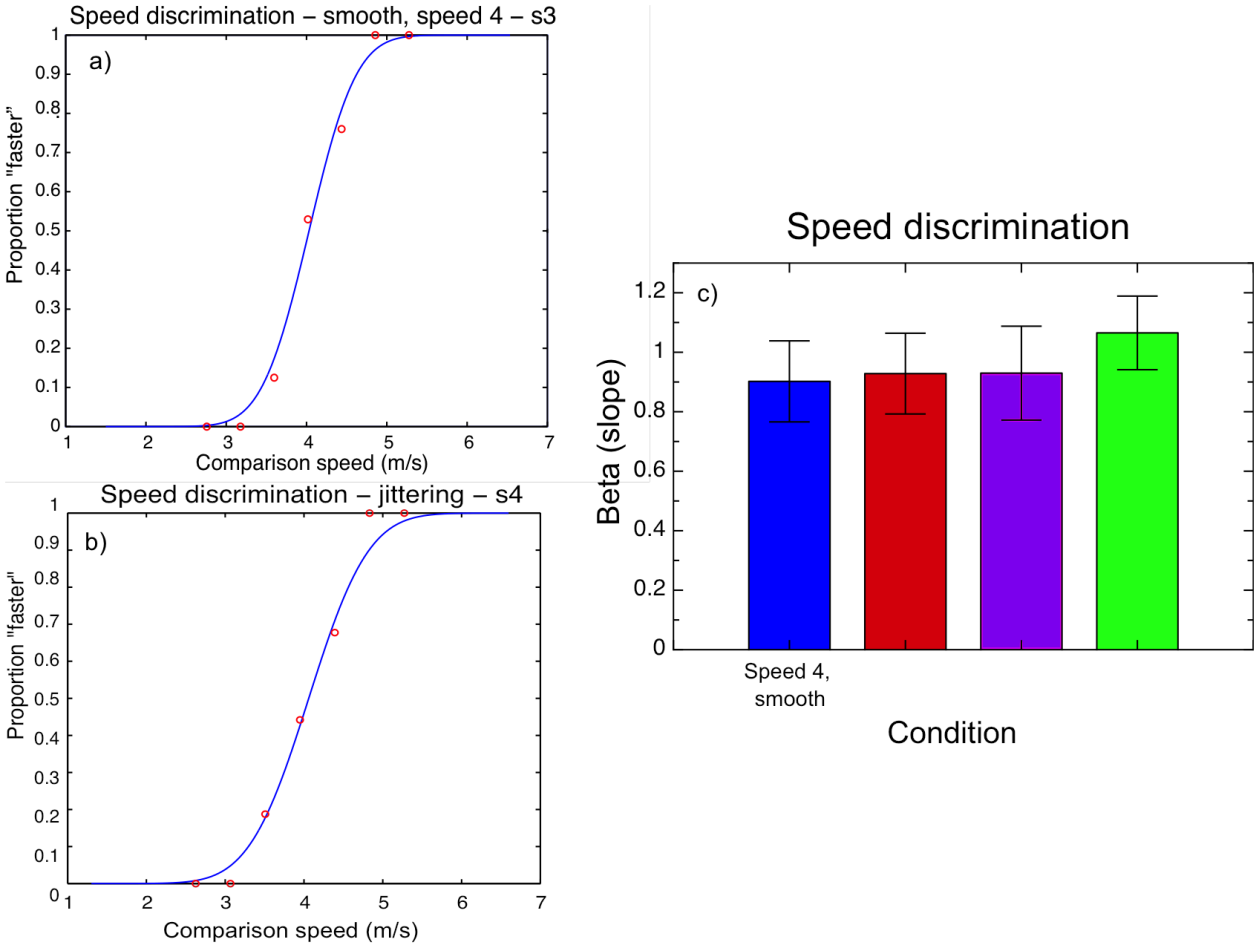
Precision estimates (betas) for discriminating the different MID stimuli. a) and b) Representative psychometric functions for two observers, pooling across 50 trials, for smooth (a) and jittering (b) MID stimuli. The functions are cumulative Gaussian fits using Maximum Likelihood Estimation. c) Mean slopes for each condition across all observers (n = 10). Error bars show +/− 1 standard error.

The results are illustrated in Figure 9. Figure 9a shows a psychometric functions for two representative subjects, which demonstrates clearly that different speeds of MID could be discriminated for each of the different types of stimuli (i.e. oscillating, jittering, and slow and fast smooth stimuli); Figure 9b shows the mean of the slopes (betas) for the 10 subjects tested. All psychometric functions showed good fits with 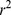 values of above .85. The mean precision, averaging across all subjects, was around 0.9 m/s for all the slower stimuli, and just over 1 m/s for the faster stimulus; none of these differed from each other statistically, F(3,27)  = 1.045, p = .389. In other words, there was no significant difference in precision for the oscillating, jittering and smooth (slower and faster) displays.

## Discussion

Previous research has shown a strong link between perceived stimulus speed and vection speed/strength: in an early review, Dichgans and Brandt note that “the perceived velocity of self-rotation during constant velocity drum rotation within a certain range is linearly related to stimulus speed (Brandt et al, 1973; Dichgans & Brandt, 1974)” [7], p. 769. In addition, Kim and Palmisano [13] show a close relationship between vection strength and the perceived speed of vection-inducing stimuli (see in particular their Figure 4). Overall, in line with these and other results from previous studies [33]–[36], [39], the results of the experiments reported here suggest that perceived MID speed does indeed play a role in vection, but less so for the jitter than for the oscillation advantage. In Experiment 1, we showed that increasing the simulated MID speed of smooth radial flow to match the perceived MID speed of oscillating displays eliminated the “oscillation advantage” for vection. However, matching perceived MID speed for jittering displays did not completely eliminate the “jitter advantage”. Interestingly, the perceived MID speed increase for jitter was significantly less than that for oscillation, although the jitter and oscillation advantages for vection were not significantly different (see Figure 8). Taken together with the findings of several recent studies [9], [12], these results appear to support the notion of separate underlying mechanisms for the two vection advantages. Below we will make the case that that these oscillation and jitter based vection advantages, though both robust and superficially similar, are likely to arise from different underlying mechanisms.

### Jitter and oscillation advantages for vection: Common mechanism or different mechanisms?

There is some previous evidence that jitter and oscillation effects may emerge from at least partially separate mechanisms. Seno, Palmisano and Ito [9] examined motion and vection aftereffects for both jittering and oscillating displays, and found distinct effects; both resulted in reduced motion aftereffects and increased vection compared to radial flow displays, but only jitter increased vection aftereffects. Although this could be related to the increased vection experienced in jittering conditions (compared to oscillating), the authors speculate that the increased VAE might be due to the adaptation of a “pure vection mechanism”, over and above lower-level motion mechanisms. According to this notion, jitter might be stimulating this pure vection mechanism to a greater extent, while oscillation may tap relatively lower-level processes through the increase in perceived speed.

Before we pursue this line of thought any further, we should first consider the possibility that perceived speed did not underlie either advantage. Logically, it could be argued that if perceived speed was greater for oscillating than for jittering stimuli, but the vection advantages were the same, then the mechanism underlying both could still be the same but something other than perceived speed. However, previous research suggests a close relationship between perceived speed and vection strength; manipulations which increase perceived speed, such as adding stereoscopic cues [35], [36] and increasing display size [37] also increase vection, while manipulations which decrease perceived speed, such as treadmill walking [40], also decrease vection. To the authors' knowledge, there are no clear reports of displays with slower perceived speeds inducing greater vection. Given the the close relationship between increases and decreases in perceived speed and increased/decreased vection for other types of stimuli, along with the results of this study, it seems likely that perceived speed does in fact play a role, albeit not a simple one.

Reduced adaptation could have been a common mechanism underlying both jitter and oscillation based vection advantages [22]. In the present study, we collected both online, continuous measures (throttle-based traces, from which we judged both latency and vection strength) and verbal, post-trial measures of subjective vection strength. These continuous measures offered useful tests of: (1) whether vection might reduce over time during each trial and (2) whether there were differential effects of adaptation for oscillating/jittering stimuli compared to smooth radial motion. However, contrary to this adaptation hypothesis, no reductions in vection were seen for any of our participants; some displayed a steady increase throughout each trial, while others showed a saturating pattern (see Figures 2 and 5). Furthermore, if jittering/oscillating displays had resulted in reduced adaptation over time, then vection should have continued to increase (or decreased less) in comparison to smooth radial motion across trials. Instead, we found that both conditions showed a linear increase in vection ratings from the first to the last trial (for both verbal and throttle ratings in Experiment 1, and for verbal ratings in Experiment 2), but there was no interaction between trial number and condition. This increase in vection over time has been observed before but never formally reported (Palmisano, personal communication); the reason for it is not entirely clear. It is possible that naïve subjects take some time to develop a response criterion for reporting vection; however, our sample included several expert observers with many years' experience of vection, and this pattern is also evident in their individual data. Another explanation might be a buildup in vection aftereffects; some researchers report a vection aftereffect in the *same direction* as vection [41], [42], specifically when the vection stimulus is followed by a blank inter-stimulus interval, as in these experiments; any aftereffects may have overlapped with subsequent vection trials, resulting in the additive effect of an increase in vection.

In principle, the jitter/oscillation advantages for vection could also both have arisen due to the misattribution of any simulated vertical acceleration to perceived MID or vection in depth. Contrary to this notion, we found that observers were able to reliably discriminate the MID speed of each type of stimulus used, and that thresholds were not different for smooth, oscillating and jittering displays. These findings suggest that observers were, in fact, able to parse out the MID from the vertical (oscillating or jittering) motion, as suggested by Kim et. al. [10], and that the increase in perceived speed in jittering and oscillating conditions was not due to misattribution of some of the vertical motion to MID. The other point that is clear from this experiment is that observers can successfully differentiate between MID stimuli regardless of the added jitter or oscillation, and with a precision finer than the perceived speed differences between smooth and oscillating stimuli, although in some cases not for jittering stimuli, since the perceived increase was less.

Thus, the past and present evidence appears to be mounting against a common mechanism for both jitter and oscillation advantages for vection. Instead, the mechanisms underlying these two advantages are likely to be either wholly or partially separate. In the current study, the strongest support for (at least partially) different mechanisms comes from the throttle data - which provided more fine-grained data on the individuals' continuous experience of vection. For instance, it was clear that for the oscillating stimuli, the speed-matched trace very closely followed the oscillating trace for all but three of the observers (see Figure 4). Of these three, only one (KD) showed an appreciable increase in vection for oscillating compared to speed-matched stimuli. However, the pattern was markedly different for the jittering stimuli (see Figure 6), where only two of the participants show a clear overlap between speed-matched and jittering stimuli, compared to slow stimuli.

In our study, in spite of the differences in perceived speed for jitter and oscillating stimuli, we did not see differences in vection strength. It is possible, since this was a between-subjects comparison, that there may have been differences between the groups in the subjective scales for vection strength. Previous research shows inconsistent findings on this point: some studies have found a greater advantage for jitter than for oscillation [9] while others have found no difference [28], as we did here; one study [12] failed to find a jitter advantage in either peripheral stationary fixation or peripheral looking conditions, although oscillation advantages were still found in these same viewing conditions. The results are somewhat difficult to compare because the temporal parameters of jitter are difficult to assess and vary considerably across studies. For instance, some update the display every frame, while others update every two or three frames (as we did); frame rates vary, amplitude of the jitter varies, and so on. It is important to note, though, that despite these variations, both jitter and oscillation advantages are remarkably robust, and in general produce very similar increases in vection despite large differences in display characteristics. Here it is notable that, though we found very similar vection advantages for jitter and oscillation (see Figure 8b–d), the increase in perceived MID speed was significantly less for jitter than for oscillation (see Figure 8a). If the perceived MID speed increase were due simply to the presence of higher temporal frequencies in the display, then it might be expected that jitter (which contains a much broader spectrum of temporal frequencies) would produce greater increases in perceived speed. Similarly, if the mechanism of the perceived MID speed increase were an increase in overall retinal motion, then jitter, due to its unpredictable nature, may often produce more retinal motion and thus higher perceived speeds. The results suggest that overall retinal motion cannot fully account for these vection advantages (in accordance with previous findings; [28]), and that oscillation and jitter advantages are likely to arise from separate mechanisms.

Since perceived speed appeared to play a stronger role in the oscillation than the jitter advantage for vection, this may be underpinned by mechanisms similar to other conditions which increase perceived speed, such as adding stereopsis [35], [36], increasing display size [37], manipulating central and peripheral spatial frequency [38], and increasing perceived depth of the stimuli [39]. If the jitter advantage is underpinned by a different mechanism, might it be a lower- or a higher-level effect? It is possible that jittering motion may indirectly stimulate vestibular mechanisms via small saccadic eye movements, which feed forward to the vestibular system via the mid-brain pathways [13], [43], whereas oscillation may increase perceived vection speed (and thus vection strength) via an increase in perceived stimulus speed, which could be modulated by relative motion or an increase in perceived depth of the display. This would suggest that jitter is tapping into a lower- rather than a higher-level mechanism. Alternatively, jitter may act to render a display more realistic or “ecological”; if this is the case, manipulating the realism of a scene should not increase perceived speed even if it increases vection. Perceived speed may thus prove a useful tool for exploring differential mechanisms underlying self-motion perception.

### Role of Perceived versus Physical Stimulus Characteristics

Another important topic area that this paper addresses is the role of perceived, compared to physical, characteristics of stimuli and their effect on self-motion perception. These topics have been widely discussed in the literature - e.g. see [34], [39], [44]. Recently, Seno and Palmisano [45] argued that perceived characteristics of the stimuli alone were insufficient to influence vection; they showed that second-order vertical oscillation added to first order (2-D) optic flow did not influence horizontal vection, although it was perceptually similar to first-order jitter (which did improve vection). This was used to argue that the oscillation advantage for vection may have a preconscious origin. However, an alternative explanation could be that first- and second-order motion systems do not interact in global motion perception [46]–[49], although see [50].

The current finding that perceived characteristics of the display (in this case, perceived MID speed) are important in determining vection strength, at least for oscillating stimuli, appears at odds with Seno and Palmisano's [45] claim. In Experiment 1, the perceived MID speed of the oscillating stimuli seemed to play a role in the subjective experience of vection, suggesting that the conscious experience of speed is important in vection. However, the finding that perceived speed seemed to be relevant for oscillation but not for jitter advantages suggests that vection speed and vection strength may not be as closely (or as simply) related as previously suggested [13].

Subjective ratings of self-motion speed have previously been shown to be closely related to ratings of vection strength; Kim and Palmisano [13] used a novel measure in which subjects were asked, after giving a verbal rating of vection strength, to adjust a smoothly-moving reference stimulus to match the perceived speed of self-motion generated by the preceding jittering stimulus, using a joystick. These joystick ratings were strongly correlated with verbal vection strength ratings. This is in keeping with earlier research [7], and provides an economical account of the findings for oscillating MID displays; vection ratings may have increased via an increase in the perceived speed of self-motion. However, since the increases in vection for jittering stimuli were similar to those for oscillating stimuli, in spite of the significantly lower perceived speed increase for jittering stimuli, this cannot provide a full explanation for the results presented here.

It is interesting that perceived speed of MID and vection measures can be shown to have a close relationship, at least for some display manipulations. However, there are still many conditions in which this relationship between perceived optic flow speed and vection has not been directly tested: would this relationship hold for vertical/horizontal linear self-motion or circular vection? Do other measures which increase vection, such as using more realistic stimuli (e.g. [19], [51], [52]), increasing object density [53], and increasing the area of motion stimulation [7], [33], [54] also increase perceived speed?

It is also likely that display manipulations which *reduce* perceived speed may also reduce vection. A recent piece of evidence [40] shows that walking on a treadmill while viewing optic flow displays produces a significant *decrease* in vection strength. Treadmill walking has also been shown by Durgin and colleagues [55], [56] to significantly decrease the perceived speed of optic flow displays. So one interesting question that might be addressed by a future study is as follows: if the treadmill walking display were matched with the perceived speed of the display viewed while standing still, would this decrease disappear? It would also be informative to explore the relationship between perceived speed and vection strength at different baseline speeds; would the effect retain a linear relationship, as in Kim and Palmisano's 2008 study [13], or would the effect lessen at very fast and/or very slow speeds?

### Conclusions

In summary, the current results show that perceived speed increases caused by oscillating and to some extent by jittering (compared to smooth) MID stimuli play a strong role in increasing perceived self-motion. The results also support the theory that oscillation and jitter advantages arise from separate mechanisms. These findings have important implications for research in the area of vection. Future studies should focus on elucidating the underlying mechanisms that might be causing both perceived speed and vection increases, and whether other methods of increasing perceived speed *without increasing retinal speed* will affect vection in a similar manner.

## Materials and Methods

### Ethics statement

The experiments were approved by the Human Ethics Committee of the University of Wollongong (approval number HE10/120). All subjects participated voluntarily and gave informed written consent. Research was conducted in accordance with the principles expressed in the Declaration of Helsinki.

### General methods

#### Participants

Participants were 10 undergraduate and graduate students (the undergraduate students received course credit for their participation), and the two authors. All had normal or corrected-to-normal vision and reported no vestibular disorders or deficits.

#### Apparatus and stimuli

The stimuli were programmed on a Mac Pro computer (Mac Pro 3.1, Quad-Core Intel Xeon 2.8 GHz) using Matlab Version R2009b and Psychtoolbox [57], [58], and displayed using a Mitsubishi Electric colour data projector (Model XD400U) back-projected onto large (1.48 m wide by 1.20 m high) screen mounted on the lab wall. Subjects viewed stimuli through black-lined viewing tube fronted by a rectangular black cardboard frame, at a distance of 1.53 m from the screen, to give a field of view of 44 degrees horizontally and 26 degrees vertically. Stimuli were random clouds consisting of 1000 blue circular dots, moving in a radially expanding fashion (see Demo Movies S1 and S2), within a virtual cloud of dots simulating a “world” 30 by 30 by 80 m. Subjects were seated on a raised chair in front of the viewing tube. Their eye-height on this chair coincided with the focus of expansion of the optic flow display. During the experiment the windowless room was darkened and any external sources of light were minimised (e.g. by turning off the external monitor, etc).

### Experiment 1.1: Speed comparison

Participants were asked to compare smooth radially-expanding flow (travelling at a simulated MID speed of 4 m/s) with radially-expanding flow that contained a vertically oscillating component (oscillation magnitude was 1/8 of the MID speed, or 0.5 m, and the frequency was 2 Hz). Each interval was a 1-second long motion display, and there was a 300 ms gap between stimulus presentations. The stimulus length of 1 second was specifically chosen as being too short to induce vection, as it is well established that it is not possible to induce illusory vection in stationary observers with display presentations under 3 seconds [7], [43]. Participants were specifically instructed to ignore the vertical motion and just match the stimuli for MID. Presentation was in a two-interval forced choice paradigm, with two randomly interleaved staircases where the speed of the smooth motion was manipulated using QUEST [59]. Responses were collected using a mouse button press, with participants responding with the left mouse button if the first interval looked faster, and the right button if the second looked faster. Each trial proceeded after a decision for the previous trial had been made. Participants each ran two blocks of two interleaved staircases comprising 25 trials each, giving a total of 100 trials for each participant. Results were then fitted with cumulative Gaussian psychometric functions using custom Matlab code, to give a value for each subject's point of subjective equality (PSE) for the speed of smooth radial flow that matched the perceived speed of the oscillating flow. This value was then used in Experiment 1.2 to present individually speed-matched displays for each participant.

### Experiment 1.2: Vection measurements for smooth, oscillating and speed-matched stimuli

Stimuli and apparatus were as described above, but displays were now presented for longer periods of time, 30 s per trial. Participants were given a throttle control device [CH Pro USB throttle] and, after being given a basic description of vection, were asked to move the throttle forwards, if and when they felt that they were moving, to rate the extent to which they felt they were moving (and specifically not the speed of their self-motion), and to move it back if they felt they were moving less or had stopped moving; the device had tactile marking points (small raised bumps at 0, 50 and 100% positions), to assist participants in rating vection strength. The computer was programmed to require the throttle to be reset to 0 before the next trial could proceed. Latency for experiencing vection was calculated as the number of seconds before the throttle value reached a threshold of 5%; throttle maximum was defined as the maximum value that the throttle reached during each 30 second trial (see Figure 4). After each trial, participants were also asked to also give a verbal rating of their vection experience, from 0 (no self-motion) to 10 (complete self-motion); this was followed by a blank period of 5 seconds to help reduce any residual effects of adaptation. Three types of trials were randomly interleaved: smooth radial motion at 4 m/s (‘slow’), smooth radial motion moving at the individually-chosen speed that matched the perceived MID speed of the oscillating stimuli (‘speed-matched’), and oscillating radial flow moving at 4 m/s and oscillating at 2 Hz, as described above (‘oscillating’). Each stimulus type was presented 4 times, and there were 2 sessions, giving a total of 8 trials per stimulus type for each participant.

### Experiment 2.1: Speed comparison for jittering stimuli

#### Participants

Participants were 8 undergraduate and graduate students (the undergraduate students received course credit for their participation), and the two authors. All had normal or corrected-to-normal vision and reported no vestibular disorders or deficits.

#### Apparatus, stimuli and procedure

These jittering radial flow displays were exactly as above, with the single exception that, instead of smooth vertical sine-wave oscillation, the stimuli were programmed to simulate random vertical viewpoint jitter, with the virtual camera moving vertically to a new, randomly-generated location every 3 frames. Since both the magnitude and the sign of this jitter varied randomly from one jittering frame to the next, it is best represented as a range of frequencies, extending from zero to the capping frequency (10 Hz) convolved with the impulse response of the display. The amplitude of this jitter was half of that reported above for the sine wave oscillation, as pilot testing showed that this jitter amplitude produced the most realistic display motion. See Demo Movie S3 for an illustration of the jittering stimulus.

### Experiment 2.2: Vection measurements for smooth, jittering and speed-matched stimuli

Experiment 2.2 was run exactly as described in Experiment 1.2, with the single exception that jittering displays (as described above) were used in place of oscillating displays.

### Experiment 3: Speed discrimination for all MID stimuli

#### Participants

Participants were 8 postgraduate students and the two authors (mean age 29.1, SD 8.65; 5 males). All had normal or corrected-to-normal vision and reported no vestibular disorders or deficits.

#### Apparatus, stimuli and procedure

The stimuli were smooth-moving, oscillating or jittering radial flow displays, exactly as described above, projected onto a large screen. The experiment was run as in Experiments 1.1 and 2.1, as a speed discrimination experiment, with the stimuli being presented in 1-second intervals interleaved with a 300-ms gap, and a mouse button used to provide the 2AFC response (which interval contained faster MID?). Two randomly-interleaved QUEST staircases were used, with 25 trials each, and each participant ran two blocks of each condition, giving a total of 100 trials for each speed discrimination. We ran 4 m/s smooth, 4 m/s jittering, 4 m/s oscillating, and 6 m/s smooth conditions - the faster condition was set between the averages of perceived speed increase for oscillating and jittering conditions, with the expectation that speed discrimination threshold might rise with either actual or perceived speed, which might throw some light on the results for vection.

## Supporting Information

Demo Movie S1 **A sample of the smooth, standard-speed MID stimulus.** Please note that speed will be approximate due to differences in frame rate and screen size.
(MOV)

Demo Movie S2 **A sample of the oscillating MID stimulus.** Please note that speed and oscillation magnitude will be approximate due to differences in frame rate and screen size.
(MOV)

Demo Movie S3 **A sample of the jittering MID stimulus.** Please note that speed and jitter magnitude will be approximate due to differences in frame rate and screen size.
(MOV)
